# Optimizing Heavy Metal Uptake in *Carpobrotus aequilaterus* Through Electrokinetic Treatment: A Comprehensive Study on Phytoremediation from Mine Tailings

**DOI:** 10.3390/toxics12120860

**Published:** 2024-11-27

**Authors:** Yasna Tapia, Osvaldo Salazar, Oscar Seguel, Jonathan Suazo-Hernández, Diego Urdiales-Flores, Humberto Aponte, Cristian Urdiales

**Affiliations:** 1 Departamento de Ingeniería y Suelos, Facultad de Ciencias Agronómicas, Universidad de Chile, Santiago 11315, Chile; yasnatapiafernandez@uchile.cl (Y.T.); osalazar@uchile.cl (O.S.); oseguel@uchile.cl (O.S.); 2 Center of Plant, Soil Interaction and Natural Resources Biotechnology, Scientific and Biotechnological Bioresource Nucleus (BIOREN-UFRO), Universidad de La Frontera, Avenida Francisco Salazar, Temuco 01145, Chile; j.suazo06@ufromail.cl; 3 Institute of Earth Surface Dynamics, University of Lausanne, 1015 Lausanne, Switzerland; diego.urdialesflores@unil.ch; 4 Laboratory of Soil Microbial Ecology and Biogeochemistry, Institute of Agri-Food, Animal and Environmental Sciences (ICA3), Universidad de O’Higgins, San Fernando 3070000, Chile; humberto.aponte@uoh.cl; 5 Centre of Systems Biology for Crop Protection (BioSaV), Universidad de O’Higgins, San Fernando 3070000, Chile; 6 Sede Vallenar, Universidad de Atacama, Av. Costanera #105, Vallenar 1612178, Chile

**Keywords:** environmental remediation, remediation efficiency, electrochemical treatment, hyperaccumulator plants, Mediterranean climate-types

## Abstract

Copper mining drives economic growth, with the global demand expected to reach 120 million metric tons annually by 2050. However, mining produces tailings containing heavy metals (HMs), which poses environmental risks. This study investigated the efficacy of phytoremediation (Phy) combined with electrokinetic treatment (EKT) to increase metal uptake in *Carpobrotus aequilaterus* grown in tailings from the Metropolitan Region of Chile. The plants were exposed to varying voltages and treatment durations. In the control (no EKT), the root metal contents were Fe (1008.41 mg/kg) > Cu (176.38 mg/kg) > Mn (103.73 mg/kg) > Zn (30.26 mg/kg), whereas in the shoots, the order was Mn (48.69 mg/kg) > Cu (21.14 mg/kg) > Zn (17.67 mg/kg) > Fe (27.32 mg/kg). The optimal EKT (15 V for 8 h) significantly increased metal uptake, with roots accumulating Fe (5997.24 mg kg^−1^) > Mn (672 mg kg^−1^) > Cu (547.68 mg kg^−1^) > Zn (90.99 mg kg^−1^), whereas shoots contained Fe (1717.95 mg kg^−1^) > Mn (930 mg kg^−1^) > Cu (219.47 mg kg^−1^) > Zn (58.48 mg kg^−1^). Although EKT enhanced plant growth and biomass, higher voltages stressed the plants. Longer treatments were more effective, suggesting that EK–Phy is a promising method for remediating metal-contaminated tailings.

## 1. Introduction

The evolution of mining has greatly contributed to the economic growth of many countries, underscoring the sector’s global importance [1]. Copper (Cu) mining, in particular, plays a crucial role in the economic development of several countries. Currently, the global Cu production capacity is approximately 22 million metric tons (MMTs) annually, led by Chile, with 5.2 MMTs, followed by Peru and Congo (2.2 MMTs each), and China (1.9 MMTs). Projections indicate that by 2050, the global Cu demand is anticipated to reach between 86 and 102 MMTs annually [2,3]. However, the extraction and processing of copper produce significant amounts of mine tailings, which account for 97% of terrestrial minerals [4]. These tailings often contain high levels of heavy metals (HMs), metalloids, and other toxic substances, posing environmental risks to soil and groundwater [5,6]. The speciation of HMs within tailings affects their bioavailability and toxicity, allowing these metals to leach into groundwater or be taken up by plants, thereby entering the food chain [7].

In Chile, approximately 764 tailings deposits were reported by 2022, with over 14% being active and approximately 84% being inactive. Most deposits are located in arid northern regions, where dry Mediterranean climates lead to local pollution from wind erosion [8]. Tailings stability depends on the structure of the deposits, including sludge, fine-grained materials, and chemicals such as silicates and sulfides [9]. When sulfide minerals in tailings interact with rainwater, sulfuric acid forms, which in turn leads to leaching that contaminates surface waters with toxic HMs, including Cu, Zn, Fe, and Pb. In northern Chile, sulphate concentrations in mine tailings range from 1243 mg L^−1^ to 2912 mg L^−1^. In comparison, natural water bodies contain 630 mg L^−1^, 2 and 250 mg L^−1^ in lakes and 230 mg L^−1^ in groundwater [10,11,12,13]. These variations illustrate the substantial impact of mining on local water quality and availability for human consumption.

HMs go into crops through contaminated irrigation water, soil absorption, or leaf deposition and accumulate in edible parts via physiological processes [14,15]. The presence of HM in the food chain poses health risks to animals and humans [16] as even trace amounts of HM can be harmful [17]. Studies have detected HM levels in various food products, including beverages, juices, and wines [18,19,20]. In Chile’s Central Zone, Cu levels in agricultural soil range from 307 mg kg^−1^ to 596 mg kg^−1^ [21]. In the Copiapó region, northern Chile, soils surrounding mine tailings have Cu concentrations between 68 mg kg^−1^ and 2116 mg kg^−1^ [22]. Currently, Chile lacks a defined soil quality standard for copper levels. In Brazil, guidelines establish an intervention value (IV) of 200 mg kg^−1^ (dry weight) [23] for Cu in agricultural soil. Copper levels in Copiapó agricultural soils often exceed this threshold. This raises concerns about potential health risks associated with elevated Cu concentrations in the region.

To reduce HM bioavailability, a wide range of remediation techniques, including chemical methods (e.g., mineral clays, nanoparticles, and biochar) and biological methods (plants, enzymes, and microorganisms), have been explored [24,25,26,27]. Phytoremediation (Phy) is a biological method in which plants are used to treat contaminated soil and water. It removes, degrades, or immobilizes harmful substances. This approach is cost-effective and sustainable. It also allows biodiversity restoration, improves aesthetics, and enhances carbon sequestration [26,27]. In northern Chile, native species such as *Atriplex nummularia*, *Prosopis tamarug*, and *Schinus molle* show promise for Cu phytostabilization [28]. In the Coquimbo Region, native/endemic plants such as *Carpobrotus aequilaterus* have demonstrated adaptability to nutrient- and water-scarce environments, whereas humic substances increase their Cu uptake [11,29]. Additionally, *C. aequilaterus* has proven effective in acid mine drainage treatment using the rhizofiltration technique [30].

Phytoremediation is a sustainable and cost-effective approach for remediating metal-contaminated soils, but its effectiveness is a standalone method. It is limited by challenges such as low metal bioavailability, phytotoxicity from high metal concentrations, and the slow pace of natural growth processes [31]. To overcome these limitations, various enhancement strategies have been developed. For instance, chelate-assisted uptake enhances metal bioavailability by forming soluble metal–chelate complexes, with synthetic chelating agents such as EDTA binding to various metal contaminants in the environment [32,33]. However, these agents pose significant environmental risks as they can mobilize heavy metals into deeper soil layers and groundwater, leading to contamination beyond the treatment zone [34,35]. Additionally, their slow degradation and high stability contribute to their persistence as environmental pollutants, sparking a growing interest in more eco-friendly, biodegradable alternatives. In response to these challenges, biodegradable chelating agents such as Ethylenediamine-N,N′-disuccinic acid (EDDS) and low molecular weight organic acids such as citric acid and glutamic diacetic acid (GLDA) have emerged as promising alternatives. These agents offer a dual benefit: they exhibit effective chelating properties while minimizing environmental impact [36]. Another technique to enhance the phytoremediation process is microbial-assisted phytoremediation, which leverages plant-microbe interactions to improve metal solubilization and absorption and offers additional potential [37,38]. These combined strategies aim to increase the efficiency and environmental sustainability of phytoremediation, particularly in addressing the challenges of highly contaminated environments such as mine tailings.

Considering that Phy is an effective process, it could struggle with highly contaminated tailings due to low nutrient levels, limited organic matter, and challenging soil conditions [39]. Electrokinetic treatment (EKT) offers a complementary approach in which direct electrical potential is applied through anodes and cathodes in tailings to mobilize contaminants toward electrodes for extraction. EKT enhances contaminant solubility and bioavailability, aiding HM uptake by plants when combined with Phy [40,41,42].

In EKT, electro-osmosis moves water toward the cathode, while electromigration directs ions toward oppositely charged electrodes, and both mechanisms improve contaminant mobility [42]. EKT induces physical and chemical changes in tailings that help to direct contaminants toward electrodes. Several studies have shown that electrodes placed in contaminated soil, combined with electrolytes to increase conductivity, improve treatment efficiency [43,44]. The tailings’ pH also influences pollutant retention or leaching, while recycling cathodic solutions to anodic areas can mitigate acidic conditions. The use of buffer solutions such as citric or acetic acid further controls the pH through complexation or chelation [45,46]. Integrating EKT with Phy benefits mine tailing remediation by applying low-intensity electric fields that increase contaminant bioavailability for plants. Electric fields can increase pollutant uptake by plants. They also redistribute heavy metal concentrations in the soil. Higher levels accumulate near the anode; in contrast, lower levels are found near the cathode [47,48].

This study aimed to evaluate a synergistic approach by integrating the contaminant uptake capacity of *C. aequilaterus* with the enhanced mobility and bioavailability of contaminants facilitated through electrical stimulation. The effectiveness of electrokinetic treatments under varying durations and applied potentials was examined, alongside an assessment of the resulting micronutrient content in *C. aequilaterus* plant tissues following the treatments.

## 2. Materials and Methods

### 2.1. Mine Tailings Samples

**Figure 1:**
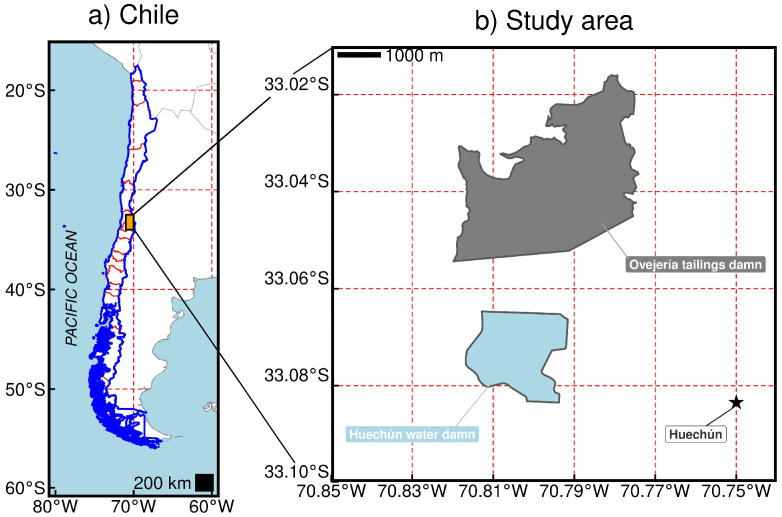
Geographical location of the study area according to (**a**) Chile reference and (**b**) the Ovejería tailings dam.

Cu mine tailing composite samples were randomly collected from the operational Ovejería tailings dam (33.05° S; 70.79° W), which is located in the surroundings of the Huechún community in the Santiago Metropolitan Region, Chile (Figure 1). The tailings samples were homogenized, air-dried, and sieved to a particle size of <2 mm for later storage in sealed plastic bins at 20 °C.

### 2.2. Determination of the Physicochemical Properties of Mine Tailings

The mine tailings were subjected to physical and chemical characterization procedures based on the methods described by Sadzawka et al. [49], including the determination of the pH of the aqueous suspension (1:2.5 *w*/*v*), electrical conductivity (EC) of the saturated extract, and OM content via the adapted Walkey–Black method. The available nitrogen (N) was measured via extraction with 2 M KCl, distillation, and titration with 0.001 M H_2_SO_4_. The Olsen method was used to determine the available phosphorus (P). For the available potassium (K) fraction, 1 M NH_4_CH_3_CO_2_ was used as an extracting agent for subsequent atomic emission spectroscopy (AES) determination via a Perkin Elmer instrument (Waltham, MA, USA). Available sulfur (SO_4_^2−^) was extracted with 0.01 M Ca(H_2_PO_4_)_2_ and measured later via turbidimetric methodology. The total contents of copper (Cu), iron (Fe), zinc (Zn), and manganese (Mn) in the tailing sample were determined via atomic absorption spectroscopy (AAS) (Perkin Elmer, Waltham, MA, USA) with previous microwave acid (HNO_3_-HF) digestion [50]. An aqueous solution containing DTPA (0.005 M), CaCl_2_ (0.01 M), and triethanolamine (0.1 M) adjusted to a pH of 7.3 was used for the extraction and assessment of available Cu, Fe, Mn, and Zn. The contents of these elements were subsequently determined using AAS. Each measurement was conducted in triplicate. The determination of soil texture was carried out using the Bouyoucos method [51]. In accordance with Stokes’s law of sedimentation [52], the Bouyoucos method uses a 5% sodium hexametaphosphate solution and a high-speed mixer to disperse soil samples efficiently.

### 2.3. Experimental Columns Setup

**Figure 2:**
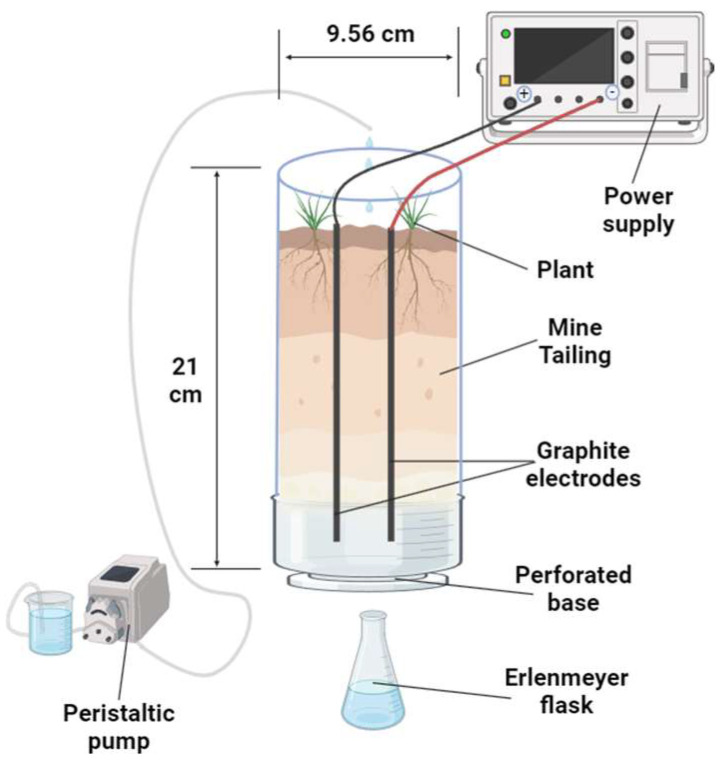
Experimental scheme of the assays.

Polyethylene columns with a height of 21 cm and an inner diameter of 9.56 cm were used (Figure 2). They were filled with mine tailings to a depth of 20 cm, ensuring uniform field bulk density. Drain holes were incorporated at the base of each column to collect the leachate. Two graphite electrodes were placed opposite each other on the column, 5 cm apart, and connected to an Ele-Tech HY3005E-2 Digital DC power supply (HYELEC, Hangzhou, China). For irrigation to provide essential macronutrients, a Hoagland-based nutrient solution was applied twice a week. Irrigation maintained the volumetric moisture content of the tailings above the experimentally determined field capacity (approximately 10% volumetric water content). A peristaltic pump connected to a reservoir facilitated the irrigation process.

Three *C. aequilaterus* plants were cultivated in each column of mine tailings, and these plants were set up through cuttings, which were directly inserted into the tailings for subsequent rooting. *C. aequilaterus* is a succulent native Chilean perennial plant used for ornamental purposes. The fruits and aerial parts of the plant are edible [29,53,54]. Additionally, *C. aequilaterus* is a recognized species capable of adapting and thriving spontaneously in mine tailings [29] because of its ability to absorb metals, metalloids, and sulphates.

This study evaluated the effectiveness of 15 V and 30 V voltage levels in enhancing HM extraction from mine tailings, which was selected on the basis of prior research highlighting their efficiency without hindering biological growth [55]. The optimization process involved preliminary trials that balanced effective HM mobilization with the ecological integrity of the medium, particularly preserving the growth of *C. chilensis*. The sustained application of voltage over seven days did not allow the development of *C. aequilaterus* environmental impacts. The treatments involved applying different voltages and durations to the mine tailings. In the first treatment (15 V-4 h), 15 volts were applied for 4 h twice a week. In the second treatment (15 V-8 h), 15 volts were applied for 8 h twice a week. In the third treatment (30 V-4 h), 30 volts were applied for 4 h on the same schedule. Finally, the fourth treatment (30 V-8 h) involved applying 30 volts for 8 h on the same schedule. Each treatment was replicated three times to ensure the reliability of the results and to optimize metal removal. For the control treatment, no voltage was applied, and the mine tailings were monitored under the same schedule without electrical intervention; the procedure was repeated three times to ensure the reliability of the results and to compare the metal removal efficiency with that of the treated samples.

The experiment lasted 45 days, during which the electrical conductivity of the leachate from each column was continuously monitored. Additionally, the soil water content was measured via a TDR probe from Campbell Scientific (Logan, UT, USA) to assess the dynamics of the water in the experimental setup.

### 2.4. Plant Growth and Biomass Accumulation

The initial area of the plant shoots (aerial part and stems) was determined before being planted in mine tailings. Throughout the growing season, we regularly measured the leaf area and root length to monitor the growth and accumulation of plant biomass in mine tailings. For leaf area determination, we used ImageJ version 1.54d image processing software.

The growth data from the plants cultivated in mine tailings were applied to a logistic growth model via Origin Pro 2019b software. The logistic growth model is defined by Equation (1):

(1) $$Y=\frac{Y_{\max}}{\left(1+e^{-k\left(t-t_{m}\right)}\right)}$$

The symmetrical sigmoid model uses Y to represent the observed foliar area values.

Where $Y_{\max}$ signifies the maximum achievable foliar area (cm^2^), k represents the growth rate (day^−1^), and t_m_ is the time of the inflection point where growth slows down (day). The inflection point occurs at 50% of the horizontal asymptote (0.5 × $Y_{\max}$) in the logistic model.

### 2.5. Analytical Determinations in Plant Tissues

The EKT + Phy experiments were conducted at the laboratory of the Faculty of Agricultural Science, University of Chile (33.56° S; 70.63° W). The laboratory maintained an average temperature of 20 °C throughout the study period, which closely resembled the mean temperature of 18.1 °C reported by the weather station in the tailings pond area. The tests were conducted in the summer between January and December.

The *C. aequilaterus* plants were preserved for 45 days. Upon completion of the test, the plants were carefully washed with distilled water. The shoots and roots were subsequently separated, and their fresh weight (FW) was subsequently determined. The plant tissues were then subjected to drying in an oven at 65 °C until a constant weight was achieved, allowing for the determination of dry biomass (DW). The dried samples were ground separately in a mill for further analysis.

For the quantification of the Cu, Fe, Mn, and Zn contents in the plant tissues, the dried samples were digested in a microwave oven with HNO_3_-HF [50] and analyzed via AAS.

### 2.6. Statistical Analysis

After evaluation of the statistical assumptions (normal distribution and homoscedasticity), one-way ANOVA with a Tukey HSD as a post hoc test was used to evaluate the individual influence of each treatment on the HM content in shoot and root tissues. The data were log10 transformed when the variables did not meet the statistical assumptions. All the statistical analyses were conducted with R statistic version 4.4.1 and the GraphPad Prism 8.0.2 software package.

## 3. Results and Discussion

### 3.1. Physicochemical Characterization of Mine Tailings

The physicochemical analysis of the Ovejería tailings sample revealed a moderately acidic pH, non-saline conditions, minimal OM content, and restricted access to essential nutrients such as N, P, and K (Table 1). The low macronutrient content (N, P, and K) creates significant challenges for reusing tailings as reforestation sites. It also hinders the establishment of self-sustaining ecosystems. This issue is widely reported in studies on Chilean tailings. Nutrient deficiencies and metal contamination further complicate efforts toward ecological restoration [11,29]. The mineral content processes resulted in significant increases in the sulphate content.

The results revealed a high total content, reflecting the mineral content in the tailings, but a relatively low bioavailable content. The physical analysis of the mine tailings revealed that sand accounted for 72.73% of the soil composition. The silt and clay fractions were low at 7.22% and 20.05%, respectively, indicating coarse-textured mine tailings with moderate water retention capacity compared with sandy topsoil [56].

### 3.2. Heavy Metal Contents in Carpobrotus aequilaterus Compartments

**Figure 3:**
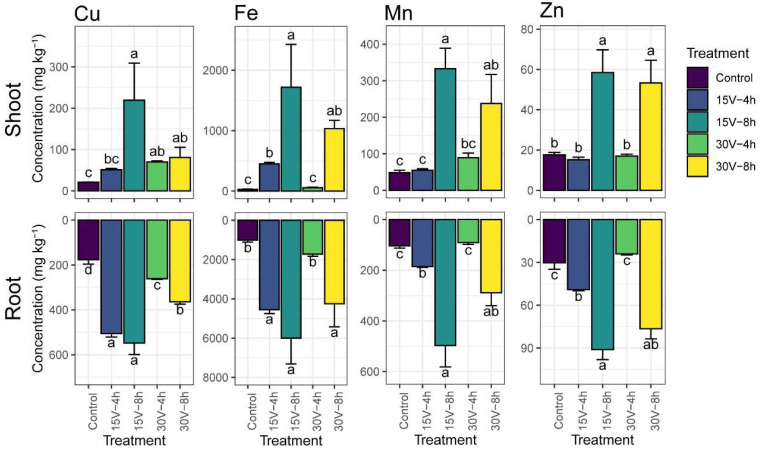
Heavy metal contents in plant tissues: Cu content; Fe content; Mn content; Zn content. Comparative analysis of the control and treatment groups: 15 V-4 h, 15 V-8 h, 30 V-4 h and 30 V-8 h. The error bar in the graph indicates the standard error of the mean, whereas different letters over the bars indicate statistically significant differences between means at *p* < 0.05.

Figure 3 displays the HM contents (Cu, Fe, Mn, and Zn) in the shoots and roots of plants subjected to different electrical treatments. These treatments included 15 V for 4 h, 15 V for 8 h, 30 V for 4 h, and 30 V for 8 h, alongside a control group. In the shoots, the Cu content in the 15 V-8 h treatment group was the highest content, significantly greater than that in the other treatment groups, whereas that in the control group was the lowest. Similarly, the Fe content was highest in the 15 V-8 h treatment and much lower in the control and 30 V-4 h treatments. Mn followed the same trend, with the 15 V-8 h treatment yielding the highest concentration and the control group the lowest. Zn showed a slightly different pattern, with both the 15 V-8 h and 30 V-8 h treatments resulting in elevated concentrations that were significantly higher than those of the control and other treatments.

In the roots, the HM content was significantly higher than that in the shoots, indicating an inverse relationship. The 15 V-8 h treatment produced the highest concentrations of Cu, Fe, and Mn, whereas significant reductions were observed in the 30 V-4 h treatment. Overall, the results suggest that electrical treatments strongly influence the distribution of metals between shoots and roots. Statistical analysis confirmed that, compared with the control, only the 15 V-8 h treatment significantly increased the Cu, Fe, Mn, and Zn contents in the roots. The Cu content was approximately three times higher than that of the control, the Fe content was six times higher, the Mn content was five times higher, and the Zn content was more than three times higher (Figure 3).

The optimized EKT at 15 V-8 h significantly increased Fe and Cu bioaccumulation within *C. aequilaterus*. The Fe content reached 5997.24 mg kg^−1^ in the roots and 1717.95 mg kg^−1^ in the shoots. These values were substantially higher than the levels recorded in prior studies with potassium humates over 120 days. In those studies, the Fe content was 2320 mg kg^−1^ in the roots and 546 mg kg^−1^ in the shoots. This study also surpassed the metal contents reported in earlier research by the same group using similar tailing samples and plant species [11].

Similarly, the optimized treatment showed superior efficacy in terms of Cu accumulation. The Cu content was 547.68 mg kg^−1^ in the roots and 219.4 mg kg^−1^ in the shoots, significantly exceeding earlier studies that reported a value of 29.3 mg kg^−1^ in the shoots [11]. These Cu levels were also comparable to those reported in *Achnatherum splendens Nevski*, which grows near mine tailings at the Ashel Cu–Zn and Kirk Tall Pb–Zn mines in Aletai, Xinjiang, China. In those areas, the Cu content was 554.2 mg kg^−1^ in the roots and 53.00 mg kg^−1^ in the shoots [57].

The Mn distribution pattern in this study differed from that reported in previous studies. The Mn content was 497.03 mg kg^−1^ in the root tissues and 332.82 mg kg^−1^ in the shoot tissues, which was significantly lower than the earlier results of 672 mg kg^−1^ in the roots and 930 mg kg^−1^ in the shoots. This variation may result from differences in soil composition, particularly the presence of potassium humates, which facilitate the formation of soluble metal complexes, thereby increasing metal bioavailability.

Zn accumulation was also slightly different. The Zn content was 90.99 mg kg^−1^ in the roots and 58.48 mg kg^−1^ in the shoots. These values were marginally lower than the earlier results in the roots (94 mg kg^−1^) and shoots (57.4 mg kg^−1^). Such differences may result from experimental conditions or genetic factors influencing Zn uptake and translocation. Zn plays essential roles in numerous enzymes and pathways, including carbohydrate and protein metabolism, auxin regulation, and membrane integrity. Zn plays essential roles as a structural component and a regulatory co-factor in more than three hundred enzymes, influencing critical pathways such as carbohydrate metabolism, which encompasses glycolysis and the citric acid cycle [58]. These pathways include the metabolism of protein and auxin, a key plant hormone responsible for growth regulation and response to light and gravity, pollen formation, maintenance of the integrity of biological membranes, and resistance to certain pathogens [59].

Several key insights emerged from the experiments. Compared with the 15 V treatment, the higher voltage (30 V) did not enhance the remediation process. Instead, stronger electric fields appeared to hinder contaminant removal by causing excessive migration toward the electrodes. This led to physiological stress in *C. aequilaterus*, reducing growth, biomass production, and nutrient extraction efficiency. Moreover, higher voltage levels may induce electrochemical reactions, degrading or transforming contaminants through oxidation or reduction.

The duration of voltage application is a critical factor influencing the effectiveness of the remediation process. Compared with the 4 h treatment, the 8 h treatment resulted in more substantial improvements in the metal content, mine tailing properties, and plant growth. Extended exposure to the electric field allowed for greater interactions between the electric field, plants, and mine tailings. This led to enhanced HM removal and improved conditions in the tailings. A longer treatment duration enabled more comprehensive redistribution and extraction of HMs. It also increased metal uptake and accumulation by plants. These processes are primarily driven by electromigration and electro-osmosis. In more detail, those mechanisms transport ionic species and fluids through the soil matrix under the influence of an electric field. This finding underscores that longer durations offer greater opportunities for achieving effective remediation outcomes.

The hierarchy of metal accumulation in plant tissues followed the order of Fe > Cu > Mn > Zn. This pattern contrasts with the bioavailability sequence in tailings, which is Fe > Cu > Zn > Mn (Table 1). This difference can be attributed to variations in metal mobility, plant uptake mechanisms, and the effects of electrokinetic treatments [40,47,48].

Electromigration and electro-osmosis enhance ion mobility within the soil matrix under an electric field. These processes preferentially mobilize Fe and Cu ions toward plant roots, aided by their high bioavailability and favorable physicochemical properties. The electric field also creates pH gradients in the soil, forming localized acidic zones that increase Fe and Cu solubility. Conversely, Mn and Zn have reduced solubilities under these conditions, limiting their mobility and plant uptake.

Plants have developed sophisticated mechanisms to acquire essential metals such as Fe and Cu. Root exudates chelate Fe ions, increasing their solubility and availability despite competition from other metals [60]. Copper uptake is supported by its vital physiological role and increased availability from electrokinetic treatments [61]. In contrast, competitive inhibition affects Mn and Zn uptake. Fe and Cu often share transport pathways with Mn and Zn, leading to preferential uptake of the former and limiting Mn and Zn accumulation in plant tissues.

Metal translocation within plant tissues follows distinct patterns. Owing to their high solubility and mobility in the vascular system, Fe and Cu are efficiently translocated from roots to shoots. In contrast, Mn and Zn exhibit lower translocation efficiencies, resulting in limited movement beyond the roots. This difference in translocation contributes to the observed hierarchy of metal accumulation in plant tissues.

In summary, metal accumulation in plant tissues is driven by electrokinetic enhancement and intrinsic plant uptake mechanisms. Owing to its high bioavailability and specialized uptake systems, Fe is dominant. Copper follows because of its physiological importance and increased availability. The limited bioavailability of Mn is due to its strong association with soil minerals, whereas Zn forms fewer mobile complexes and competitively inhibits Fe and Cu. Understanding these dynamics is essential for optimizing phytoremediation strategies and improving nutrient management in agricultural systems.

The experiments demonstrated the effectiveness of combining electrokinetic (EK) and phytoremediation (Phy) treatments for remediating mine tailings. These treatments effectively removed contaminants and improved environmental conditions. Key findings emphasized the importance of optimizing both voltage and duration to enhance treatment efficacy. The study revealed that 15 V was more effective than 30 V for contaminant removal without disrupting plant physiology. Prolonged exposure to an electric field significantly improved remediation outcomes. Longer treatment durations resulted in improved contaminant extraction and enhanced plant metal uptake. These findings highlight the potential of extended treatments to achieve more comprehensive and effective remediation results.

### 3.3. Plant Growth

**Figure 4:**
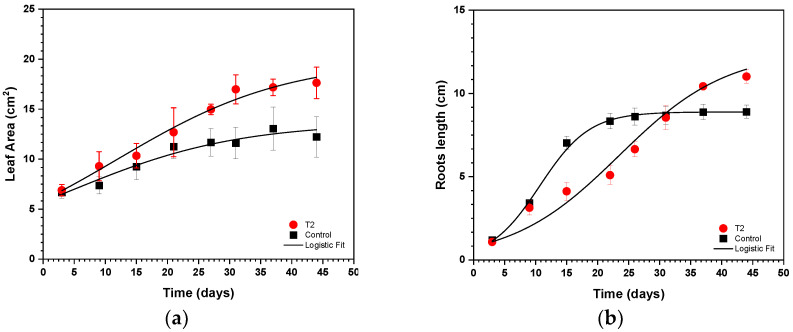
Variations in the leaf area of the designated *C. aequilaterus* shoots (**a**) and root length (**b**) during their expansion were noted for both the control group and the optimal treatment conditions (15 V-8 h). The research included four plants, with the vertical bars depicted on the graph indicating the standard error of the mean. The associations among the observed data points were delineated via a logistic growth model.

The application of the logistic growth model provided valuable insights into plant growth dynamics. Figure 4 shows the logistic root growth curves and the experimental determination of the leaf area for the optimal EK treatment (15 V-8 h) and the control group. Table 2 shows the fitted parameters of the logistic growth model.

In the control group, the model parameters gradually increased. The maximum foliar area reached 13.63 cm^2^, with an inflection point at 4.291 days. The goodness-of-fit indicators confirmed that the model effectively represented the growth dynamics of the control group. In contrast, the 15 V-8 h treatment resulted in a distinct growth trajectory. The parameters indicated a greater maximum foliar area of 19.93 cm^2^ and a later inflection point at 11.86 days. Notably, the model’s predictions closely matched the actual growth data, as evidenced by the high goodness-of-fit values.

The logistic growth model analysis of root growth also revealed significant findings. In the control group, root growth followed a well-fit logistic curve with parameters of Y_max_ = 8.89 cm, t_m_ = 10.66 days, and k = 0.2495 day^−1^. This alignment demonstrated a strong correlation (R^2^ = 0.9985) between the model and observed growth. Conversely, the 15 V-8 h treatment presented altered dynamics, with Y_max_ = 12.54 cm, t_m_ = 23.46 day, and k = 0.1148 day^−1^. Although the model fit was slightly less accurate, as reflected by a greater reduction in the chi-square value of 2.6585, the R^2^ value remained high at 0.9979, indicating a consistent growth trend.

The integration of EKT + Phy requires careful consideration of environmental risks. The introduction of an electric field can alter the soil pH, creating extremely acidic conditions near the anode and alkaline conditions near the cathode. These pH extremes negatively impact plant growth and microbial activity by causing nutrient deficiencies or toxicity. Additionally, electrical potential can significantly influence microbial and enzymatic activities [62]. Key enzymes such as urease, invertase, and phosphatase, which are critical for nutrient cycling and organic matter decomposition, may be adversely affected. Thus, the overall effectiveness of phytoremediation may be reduced.

The application of an electric field to mine tailings also generates heat due to the resistivity of the tailings [63,64], which can alter the physical properties of the tailings. This creates an unfavorable environment for both plants and soil. This research aims to optimize the use of EKT + Phy while minimizing environmental risk. Controlled electrical potentials between 15 V and 30 V were applied for 4 or 8 h, twice weekly, to avoid extreme acidic or alkaline conditions in the tailing soil profile. Additionally, this study explored succulent and shrubby plants that are tolerant to harsh conditions, aiding in contaminant degradation or stabilization. These strategies aim to increase the sustainability and effectiveness of phytoremediation technologies for managing mine tailings.

In summary, the results demonstrate that electrokinetic treatment significantly enhances metal uptake and accelerates plant growth. These findings establish it as a promising tool for optimizing phytoremediation in mine tailing reclamation. Careful calibration of electrokinetic parameters can improve remediation efficiency and plant health. These findings support the potential of this approach as a viable strategy for the ecological restoration of contaminated sites.

## 4. Conclusions

The combined use of electrokinetic treatment (EKT) and phytoremediation (Phy) represents a promising method for the remediation of metal-contaminated mine tailings, as evidenced by enhanced metal uptake and improved plant growth. This study demonstrated that applying a 15 V potential for 8 h significantly increased HM accumulation within plant tissues while simultaneously increasing plant growth rates, suggesting that this electrokinetic–phytoremediation approach could be an effective solution for ecological restoration in contaminated environments. However, our findings underscore that the effectiveness of this method is influenced by key factors, including the composition of tailings, pH levels, moisture content, and choice of plant species, each of which has a direct effect on the remediation process and growth dynamics.

Our results highlight the need for precise optimization of electrokinetic parameters, such as voltage application and duration, to maximize both metal uptake and plant health. The enhanced root dynamics and biomass accumulation observed in this study point to a potentially synergistic effect of EKT and Phy, which warrants further investigation to elucidate the underlying mechanistic interactions. For example, understanding how electric fields influence root architecture and growth kinetics could offer valuable insights into plant-based reclamation techniques and inform targeted strategies for other metal-polluted environments.

Future studies should also aim to evaluate the long-term ecological impacts of electrokinetic phytoremediation, including any possible effects on soil microbial communities, which play a crucial role in sustainable ecosystem recovery. Additionally, research into the scalability of this approach under field conditions is essential for assessing its feasibility in diverse mining environments, where soil properties and environmental conditions may vary widely. Further research will enable the development of more effective, site-specific strategies to enhance metal uptake and strengthen plant resilience, as well as to reduce the environmental footprint of mining activities. This knowledge will play a crucial role in designing sustainable, large-scale restoration techniques that prioritize ecological aspects.

## Figures and Tables

**Table 1:** Physicochemical characterization of the Ovejería mine tailings.

Parameter	Value
pH	5.80 ± 0.055
EC (mS cm^−1^)	2.74 ± 0.04
Organic matter (%)	0.55 ± 0.04
Available N (mg kg^−1^)	3.73 ± 0.47
Available P (mg kg^−1^)	1.64 ± 0.02
Available K (mg kg^−1^)	6.23 ± 0.17
Available SO_4_^2−^ (mg kg^−1^)	410 ± 12
Bioavailable Cu (mg kg^−1^)	28.1 ± 1.04
Bioavailable Fe (mg kg^−1^)	307 ± 9.23
Bioavailable Mn (mg kg^−1^)	1.73 ± 0.23
Bioavailable Zn (mg kg^−1^)	4.37 ± 0.31
Total Cu (mg kg^−1^)	1924 ± 119
Total Fe (mg kg^−1^)	8769 ± 468
Total Mn (mg kg^−1^)	187 ± 3.58
Total Zn (mg kg^−1^)	92.6 ± 10.2
Bulk density (g cm^−3^)	1.67
Sand (%)	72.73
Silt (%)	7.22
Clay (%)	20.05

**Table 2:** Fitted parameters of the logistic growth model for the shoots and roots of *Carpobrotus aequilaterus* in the control and 15 V-8 h groups.

	$Y_{\max}$ (cm^2^)	$t_{m}$ (day)	K (day^−1^)	Reduced Chi-Sqr	R^2^
Shoots					
Control	13.63 ± 1.349	4.291 ± 2.737	0.07405 ± 0.02037	0.2466	0.9647
15 V-8 h	19.93 ± 1.176	11.86 ± 2.033	0.07304 ± 0.00834	0.2186	0.9939
Roots					
Control	8.889 ± 0.1303	10.66 ± 0.2453	0.2495 ± 0.01121	0.3277	0.9985
15 V-8 h	12.54 ± 0.957	23.46 ± 2.497	0.1148 ± 0.01054	2.6585	0.9979

## Data Availability

Data are contained within the article.

## References

[1] Candeias C., Ávila P., Coelho P., Teixeira J.P. (2019). Mining Activities: Health Impacts. Encyclopedia of Environmental Health.

[2] USGS Copper Statistics and Information. https://pubs.usgs.gov/periodicals/mcs2023/mcs2023-copper.pdf.

[3] Seck G.S., Hache E., Bonnet C., Simoën M., Carcanague S. (2020). Copper at the Crossroads: Assessment of the Interactions between Low-Carbon Energy Transition and Supply Limitations. Resour. Conserv. Recycl..

[4] Edraki M., Baumgartl T., Manlapig E., Bradshaw D., Franks D.M., Moran C.J. (2014). Designing Mine Tailings for Better Environmental, Social and Economic Outcomes: A Review of Alternative Approaches. J. Clean. Prod..

[5] Ramirez M., Massolo S., Frache R., Correa J.A. (2005). Metal Speciation and Environmental Impact on Sandy Beaches Due to El Salvador Copper Mine, Chile. Mar. Pollut. Bull..

[6] Stauber J.L., Andrade S., Ramirez M., Adams M., Correa J.A. (2005). Copper Bioavailability in a Coastal Environment of Northern Chile: Comparison of Bioassay and Analytical Speciation Approaches. Mar. Pollut. Bull..

[7] Zhang X., Yang H., Cui Z. (2017). Migration and Speciation of Heavy Metal in Salinized Mine Tailings Affected by Iron Mining. Water Sci. Technol..

[8] Gerding J., Novoselov A.A., Morales J. (2021). Climate and Pyrite: Two Factors to Control the Evolution of Abandoned Tailings in Northern Chile. J. Geochem. Explor..

[9] Ritcey G.M. (2005). Tailings Management in Gold Plants. Hydrometallurgy.

[10] Santibañez C., De La Fuente L.M., Bustamante E., Silva S., León-Lobos P., Ginocchio R. (2012). Potential Use of Organic- and Hard-Rock Mine Wastes on Aided Phytostabilization of Large-Scale Mine Tailings under Semiarid Mediterranean Climatic Conditions: Short-Term Field Study. Appl. Environ. Soil Sci..

[11] Tapia Y., Bustos P., Salazar O., Casanova M., Castillo B., Acuña E., Masaguer A. (2017). Phytostabilization of Cu in Mine Tailings Using Native Plant Carpobrotus Aequilaterus and the Addition of Potassium Humates. J. Geochem. Explor..

[12] Nieva N.E., Borgnino L., García M.G. (2018). Long Term Metal Release and Acid Generation in Abandoned Mine Wastes Containing Metal-Sulphides. Environ. Pollut..

[13] UNEP G. (1990). Water Data Summary 1985–1987.

[14] Kumar S., Prasad S., Yadav K.K., Shrivastava M., Gupta N., Nagar S., Bach Q.V., Kamyab H., Khan S.A., Yadav S. (2019). Hazardous Heavy Metals Contamination of Vegetables and Food Chain: Role of Sustainable Remediation Approaches—A Review. Environ. Res..

[15] Liu X., Song Q., Tang Y., Li W., Xu J., Wu J., Wang F., Brookes P.C. (2013). Human Health Risk Assessment of Heavy Metals in Soil–Vegetable System: A Multi-Medium Analysis. Sci. Total Environ..

[16] Hosseinniaee S., Jafari M., Tavili A., Zare S., Cappai G. (2023). Investigating Metal Pollution in the Food Chain Surrounding a Lead-Zinc Mine (Northwestern Iran); an Evaluation of Health Risks to Humans and Animals. Environ. Monit. Assess..

[17] EPA U.S. (1996). Integrated Risk Information System (IRIS) [Online Electronic Data File].

[18] Wang Z., Jackson L.S., Jablonski J.E. (2017). Factors Affecting the Levels of Heavy Metals in Juices Processed with Filter Aids. J. Food Prot..

[19] Ryan R. (2014). Safety of Food and Beverages: Soft Drinks and Fruit Juices. Encycl. Food Saf..

[20] Asuku A.O., Ayinla M.T., Ajibare A.J., Adeyemo M.B., Adeyemo R.O. (2024). Heavy Metals and Emerging Contaminants in Foods and Food Products Associated with Neurotoxicity. Emerging Contaminants in Food and Food Products.

[21] Neaman A., Reyes L., Trolard F., Bourrié G., Sauvé S. (2009). Copper Mobility in Contaminated Soils of the Puchuncaví Valley, Central Chile. Geoderma.

[22] Carkovic A.B., Calcagni M.S., Vega A.S., Coquery M., Moya P.M., Bonilla C.A., Pastén P.A. (2016). Active and Legacy Mining in an Arid Urban Environment: Challenges and Perspectives for Copiapó, Northern Chile. Environ. Geochem. Health.

[23] CETESB (2005). Companhia de Tecnologia de Saneamento Ambiental Decisão de Diretoria.

[24] Munir M.A.M., Irshad S., Yousaf B., Ali M.U., Dan C., Abbas Q., Liu G., Yang X. (2021). Interactive Assessment of Lignite and Bamboo-Biochar for Geochemical Speciation, Modulation and Uptake of Cu and Other Heavy Metals in the Copper Mine Tailing. Sci. Total Environ..

[25] Mujtaba Munir M.A., Liu G., Yousaf B., Ali M.U., Abbas Q., Ullah H. (2020). Synergistic Effects of Biochar and Processed Fly Ash on Bioavailability, Transformation and Accumulation of Heavy Metals by Maize (*Zea mays* L.) in Coal-Mining Contaminated Soil. Chemosphere.

[26] Doku E.T., Sylverken A.A., Belford J.D.E. (2024). Rhizosphere Microbiome of Plants Used in Phytoremediation of Mine Tailing Dams. Int. J. Phytoremediat..

[27] Yongpisanphop J., Babel S., Kruatrachue M., Pokethitiyook P. (2017). Phytoremediation Potential of Plants Growing on the Pb-Contaminated Soil at the Song Tho Pb Mine, Thailand. Soil Sediment Contam. Int. J..

[28] Lam E.J., Cánovas M., Gálvez M.E., Montofré Í.L., Keith B.F., Faz Á. (2017). Evaluation of the Phytoremediation Potential of Native Plants Growing on a Copper Mine Tailing in Northern Chile. J. Geochem. Explor..

[29] Orchard C., León-Lobos P., Ginocchio R. (2009). Phytostabilization of Massive Mine Wastes with Native Phytogenetic Resources: Potential for Sustainable Use and Conservation of the Native Flora in North-Central Chile. Cienc. Investig. Agrar..

[30] Tapia Y., Salazar O., Joven A., Castillo B., Urdiales C., Garcia A., Ihle C., Acuña E. (2024). Evaluation of Sulfate Rhizofiltration by *Carpobrotus chilensis* for Treating Mining Waters. Int. J. Phytoremediat..

[31] Adeoye A.O., Adebayo I.A., Afodun A.M., Ajijolakewu K.A. (2022). Benefits and Limitations of Phytoremediation: Heavy Metal Remediation Review. Phytoremediation.

[32] Shahid M., Austruy A., Echevarria G., Arshad M., Sanaullah M., Aslam M., Nadeem M., Nasim W., Dumat C. (2014). EDTA-Enhanced Phytoremediation of Heavy Metals: A Review. Soil Sediment Contam. Int. J..

[33] Lim J.M., Salido A.L., Butcher D.J. (2004). Phytoremediation of Lead Using Indian Mustard (*Brassica juncea*) with EDTA and Electrodics. Microchem. J..

[34] Guo J.K., Lv X., Jia H.L., Hua L., Ren X.H., Muhammad H., Wei T., Ding Y. (2020). Effects of EDTA and Plant Growth-Promoting Rhizobacteria on Plant Growth and Heavy Metal Uptake of Hyperaccumulator Sedum Alfredii Hance. J. Environ. Sci..

[35] Beiyuan J., Tsang D.C.W., Bolan N.S., Baek K., Ok Y.S., Li X.D. (2018). Interactions of Food Waste Compost with Metals and Metal-Chelant Complexes during Soil Remediation. J. Clean. Prod..

[36] Yin F., Li J., Wang Y., Yang Z. (2024). Biodegradable Chelating Agents for Enhancing Phytoremediation: Mechanisms, Market Feasibility, and Future Studies. Ecotoxicol. Environ. Saf..

[37] Aponte H., Sulbaran-Bracho Y., Mondaca P., Vidal C., Pérez R., Meier S., Cornejo P., Rojas C. (2023). Biochemical, Catabolic, and PGP Activity of Microbial Communities and Bacterial Strains from the Root Zone of Baccharis Linearis in a Mediterranean Mine Tailing. Microorganisms.

[38] Novo L.A.B., Castro P.M.L., Alvarenga P., da Silva E.F. (2018). Plant Growth–Promoting Rhizobacteria-Assisted Phytoremediation of Mine Soils. Bio-Geotechnologies for Mine Site Rehabilitation.

[39] Wang L., Ji B., Hu Y., Liu R., Sun W. (2017). A Review on in Situ Phytoremediation of Mine Tailings. Chemosphere.

[40] Cang L., Wang Q.Y., Zhou D.M., Xu H. (2011). Effects of Electrokinetic-Assisted Phytoremediation of a Multiple-Metal Contaminated Soil on Soil Metal Bioavailability and Uptake by Indian Mustard. Sep. Purif. Technol..

[41] Chen Y., Dong M., Lyu P., Wang A., Wang H., Li J. (2023). Analysis of Metal(Loid) Pollution and Possibilities of Electrokinetic Phytoremediation of Abandoned Coking Plant Soil. Sci. Total Environ..

[42] Cameselle C., Reddy K.R. (2012). Development and Enhancement of Electro-Osmotic Flow for the Removal of Contaminants from Soils. Electrochim. Acta.

[43] Stegmann R., Brunner G., Calmano W., Matz G. (2001). Treatment of Contaminated Soil.

[44] Robles I., Serrano T., Pérez J.J., Hernández G., Solís S., García R., Pi T., Bustos E. (2014). Influence of EDTA on the Electrochemical Removal of Mercury (II) in Soil from San Joaquín, Querétaro, México. J. Mex. Chem. Soc..

[45] Popescu M., Rosales E., Sandu C., Meijide J., Pazos M., Lazar G., Sanromán M.A. (2017). Soil Flushing and Simultaneous Degradation of Organic Pollutants in Soils by Electrokinetic-Fenton Treatment. Process Saf. Environ. Prot..

[46] Kim G.N., Jung Y.H., Lee J.J., Moon J.K., Jung C.H. (2008). An Analysis of a Flushing Effect on the Electrokinetic-Flushing Removal of Cobalt and Cesium from a Soil around Decommissioning Site. Sep. Purif. Technol..

[47] Cameselle C., Chirakkara R.A., Reddy K.R. (2013). Electrokinetic-Enhanced Phytoremediation of Soils: Status and Opportunities. Chemosphere.

[48] Chirakkara R.A., Reddy K.R., Cameselle C. (2015). Electrokinetic Amendment in Phytoremediation of Mixed Contaminated Soil. Electrochim. Acta.

[49] Sadzawka A., Carrasco M.A., Grez R., Mora M.L., Flores H., Neaman A. (2006). Métodos de Análisis de Suelos Recomendados Para Los Suelos de Chile: Revisión 2006. https://biblioteca.inia.cl/items/9ec1aab6-f9aa-4e4b-b2c5-28d1fed798c3.

[50] United States Environmental Protection Agency (1996). Method 3052: Microwave Assisted Acid Digestion of Siliceous and Organically Based Matrices.

[51] Bouyoucos G.J. (1962). Hydrometer Method Improved for Making Particle Size Analyses of Soils1. Agron. J..

[52] Jury W.A., Horton R. (2004). Soil Physics.

[53] Villagrán C., Castro Rojas M.V. (2004). Ciencia Indígena de Los Andes Del Norte de Chile: Programa Interdisciplinario de Estudios En Biodiversidad (PIEB), Universidad de Chile.

[54] Rodriguez R., Marticorena C., Alarcón D., Baeza C., Cavieres L., Finot V.L., Fuentes N., Kiessling A., Mihoc M., Pauchard A. (2018). Catálogo de Las Plantas Vasculares de Chile. Gayana Bot..

[55] Hansen H.K., Lamas V., Gutierrez C., Nuñez P., Rojo A., Cameselle C., Ottosen L.M. (2013). Electro-Remediation of Copper Mine Tailings. Comparing Copper Removal Efficiencies for Two Tailings of Different Age. Min. Eng..

[56] Robson T., Golos P.J., Stevens J., Reid N. (2018). Enhancing Tailings Revegetation Using Shallow Cover Systems in Arid Environments: Hydrogeochemical, Nutritional, and Ecophysiological Constraints. Land. Degrad. Dev..

[57] Liu Z., Hamuti A., Abdulla H., Zhang F., Mao X. (2016). Accumulation of Metallic Elements by Native Species Thriving in Two Mine Tailings in Aletai, China. Environ. Earth Sci..

[58] Zeng H., Wu H., Yan F., Yi K., Zhu Y. (2021). Molecular Regulation of Zinc Deficiency Responses in Plants. J. Plant Physiol..

[59] Sadeghzadeh B., Rengel Z. (2011). Zinc in Soils and Crop Nutrition. The Molecular and Physiological Basis of Nutrient Use Efficiency in Crops.

[60] Clemens S., Weber M. (2016). The Essential Role of Coumarin Secretion for Fe Acquisition from Alkaline Soil. Plant Signal Behav..

[61] Pandey N. (2018). Role of Plant Nutrients in Plant Growth and Physiology. Plant Nutrients and Abiotic Stress Tolerance.

[62] Cang L., Zhou D.M., Wang Q.Y., Fan G.P. (2012). Impact of Electrokinetic-Assisted Phytoremediation of Heavy Metal Contaminated Soil on Its Physicochemical Properties, Enzymatic and Microbial Activities. Electrochim. Acta.

[63] Wu C., Fan C., Xie Q. (2012). Study on Electrokinetic Remediation of PBDEs Contaminated Soil. Adv. Mater. Res..

[64] Fu R., Wen D., Xia X., Zhang W., Gu Y. (2017). Electrokinetic Remediation of Chromium (Cr)-Contaminated Soil with Citric Acid (CA) and Polyaspartic Acid (PASP) as Electrolytes. Chem. Eng. J..

